# NOT READY FOR PRIME TIME: A FAILED STUDY TESTING A TECHNOLOGICAL INTERVENTION IN NURSING HOME CARE

**DOI:** 10.1093/geroni/igz038.2834

**Published:** 2019-11-08

**Authors:** Kristine N Williams, Clarissa Shaw

**Affiliations:** 1 University of Kansas Medical Center, Kansas City, Kansas, United States; 2 University of Iowa, College of Nursing, Iowa City, Iowa, United States

## Abstract

This pilot-study tested an adaptation of a successful family caregiver telehealth support intervention in nursing home settings. The goal was to support staff in managing challenging behaviors of residents with dementia. Nursing homes were provided with an iPad mini equipped with a special application to video record staff-resident challenging care situations. Videos were uploaded to a secure site for review by dementia care experts who provided feedback to improve care. Despite efforts to engage staff, only four videos were submitted. Factors including privacy, workload, and fear of documenting abuse contributed to implementation failure. The same intervention was successfully implemented in the home setting; differences in engagement and utilization will be discussed. Understanding the unique environments of nursing home dementia care is needed to successfully implement technology-based interventions to improve care. Evaluation of factors predicting the failure of this intervention may inform future research.

